# Sahel rainfall strength and onset improvements due to more realistic Atlantic cold tongue development in a climate model

**DOI:** 10.1038/s41598-018-20904-1

**Published:** 2018-02-07

**Authors:** S. Steinig, J. Harlaß, W. Park, M. Latif

**Affiliations:** 1 GEOMAR Helmholtz Centre for Ocean Research Kiel, Wischhofstr. 1-3, 24148 Kiel, Germany; 2 Kiel University, Kiel, Germany

**Keywords:** Atmospheric dynamics, Physical oceanography

## Abstract

The simulation of Sahel rainfall and its onset during the West African Monsoon (WAM) remains a challenge for current state-of-the-art climate models due to their persistent biases, especially in the tropical Atlantic region. Here we show that improved representation of Atlantic Cold Tongue (ACT) development is essential for a more realistic seasonal evolution of the WAM, which is due to a further inland migration of the precipitation maximum. The observed marked relationship between ACT development and Sahel rainfall onset only can be reproduced by a climate model, the Kiel Climate Model (KCM), when sufficiently high resolution in its atmospheric component is employed, enabling enhanced equatorial Atlantic interannual sea surface temperature variability in the ACT region relative to versions with coarser atmospheric resolution. The ACT/Sahel rainfall relationship in the model critically depends on the correct seasonal phase-locking of the interannual variability rather than on its magnitude. We compare the KCM results with those obtained from climate models participating in the Coupled Model Intercomparison Project phase 5 (CMIP5).

## Introduction

The Sahel has experienced severe and widespread droughts during the 1970s and 1980s that led to dramatic consequences for the mainly rain-fed agriculture. These were the most significant climatic events at the continental scale during the twentieth century^1^ and have motivated extensive research on the causes of the observed rainfall variability over the last decades.

Sahel rainfall is inevitably linked to the annual cycle of the West African Monsoon (WAM). The associated northward migration of the Intertropical Convergence Zone (ITCZ) is the result of a complicated interaction between the Saharan heat low developing over the warming continent and the cooling ocean associated with the Atlantic Cold Tongue (ACT) development^2^. The maximum continental warming follows the solar heating northward, whereas the maximum cooling of the ocean surface, driven by meridional and zonal wind stress, is geographically fixed just south of the Equator. These different physical processes combined with land-air interactions^3,4^ lead to a more step-wise than gradual onset of the WAM^5^. The influence of slowly varying climate system components, e.g. the tropical Atlantic Ocean, on the WAM evolution potentially allows useful predictions of its variability^6^. Sahel rainfall from July to September is initiated by the sudden shift of the precipitation maximum from the Gulf of Guinea at 5°N to the southern Sahel around 10°N and accounts for about 80% of the annual precipitation^7^.

**Figure 1:**
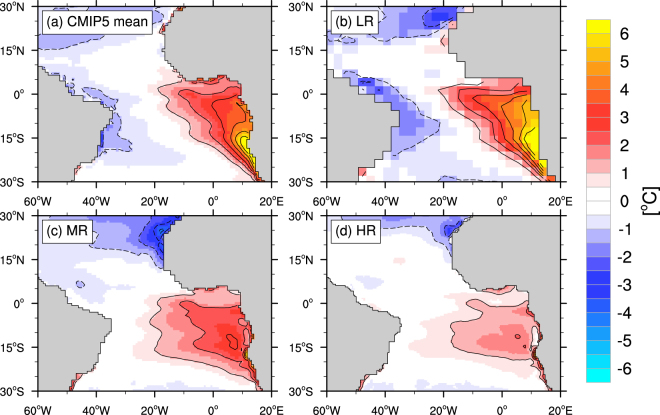
SST biases against OISSTv2 for the months July to August. Values are in °C (contour line interval is 1 °C). (**a**) CMIP5 ensemble-mean from the historical runs for the years 1982–2005, (**b**) LR (T42L31), (**c**) MR (T159L62), (**d**) HR (T255L62). Maps are created with the NCAR Command Language (NCL) version 6.4.0^41^.

Given the limited observations in this region, climate models would be an ideal tool to identify the causes of past variability and assess the future evolution. But persistent biases especially in the tropical Atlantic region challenge the applicability of current climate models in addressing those questions. Nearly all state-of-the-art climate models suffer from serious biases in precipitation, surface winds and temperatures in this region^8,9^. One of the most apparent shortcomings is the too warm sea surface temperatures (SSTs) in the eastern tropical Atlantic during boreal spring and summer^10^ (Fig. 1a) and a corresponding reversed equatorial zonal SST gradient. In contrast to the tropical Pacific, most models are not able to reproduce the seasonal cold tongue evolution in the tropical Atlantic south of the equator. Moreover, the errors in the mean state strongly bias the interannual variability in the models^11^. Only minor progress has been made in the recent years in reducing these biases^12^.

Furthermore, possible implications for the precipitation on adjacent continents arising from these biases are poorly understood and surprisingly rarely discussed^13^. Due to the strong coupling of tropical Atlantic SSTs to the meridional atmospheric pressure gradient and the seasonal evolution of the WAM, the biases severely limit the application of current coupled models for questions regarding Sahel rainfall, both for explaining the past and predicting the future^14^. In fact, climate model projections of Sahel rainfall for the twenty-first century are highly uncertain, disagreeing even on the sign of future trends^15–17^.

Harlaß *et al*.^18,19^ greatly reduced the strength and extent of the tropical Atlantic warm SST bias in the Kiel Climate Model (KCM) by a simultaneous increase of horizontal and vertical atmospheric model resolution leading among others to enhanced surface wind stress. A large part of the wind stress biases at lower resolution arise from insufficient meridional and vertical zonal momentum transport in the atmosphere, and the misrepresentation of the orography surrounding the tropical Atlantic in the spectral atmospheric component^20^.

Here we follow up the work of Harlaß *et al*.^18,19^ and investigate how different representations of the tropical Atlantic mean state, seasonal cycle and interannual variability influence Sahel rainfall in different configurations of the KCM (see methods). In contrast to applying a fixed (state-independent) bias correction, we are able to analyze the full dynamic response of the WAM to an improved tropical Atlantic sector climate and its variability.

## Results

The common limitations in simulating tropical Atlantic boreal summer SSTs are obvious both in the CMIP5 ensemble-mean (Fig. 1a) as well as in the KCM low-resolution configuration LR (Fig. 1b). The largest deficit is a strong warm bias in the eastern part of the basin with maximum values in the upwelling regions off Southwest Africa. The warm bias persists throughout the year with maximum errors exceeding 6 °C at 20°S during boreal spring and summer. The western equatorial region is marked by colder than observed SSTs leading to absolute temperatures that are erroneously lower than those in the eastern part. The simultaneous increase of horizontal and vertical atmospheric resolution in MR (Fig. 1c) greatly reduces the warm bias in the east to below 3 °C and shifts its core slightly away from the coast. The stronger surface wind stress responsible for reducing the warm bias in the Benguela Current region^18^ also increases upwelling of colder waters in the Canary Current in the northeastern tropical Atlantic. The resulting reduced SSTs lead to an amplified meridional dipole bias pattern along the African coastline. The further increase of horizontal resolution in HR (Fig. 1d) reduces both the biases in the southeastern and northeastern tropical Atlantic to maximum values below 2 °C.

**Figure 2:**
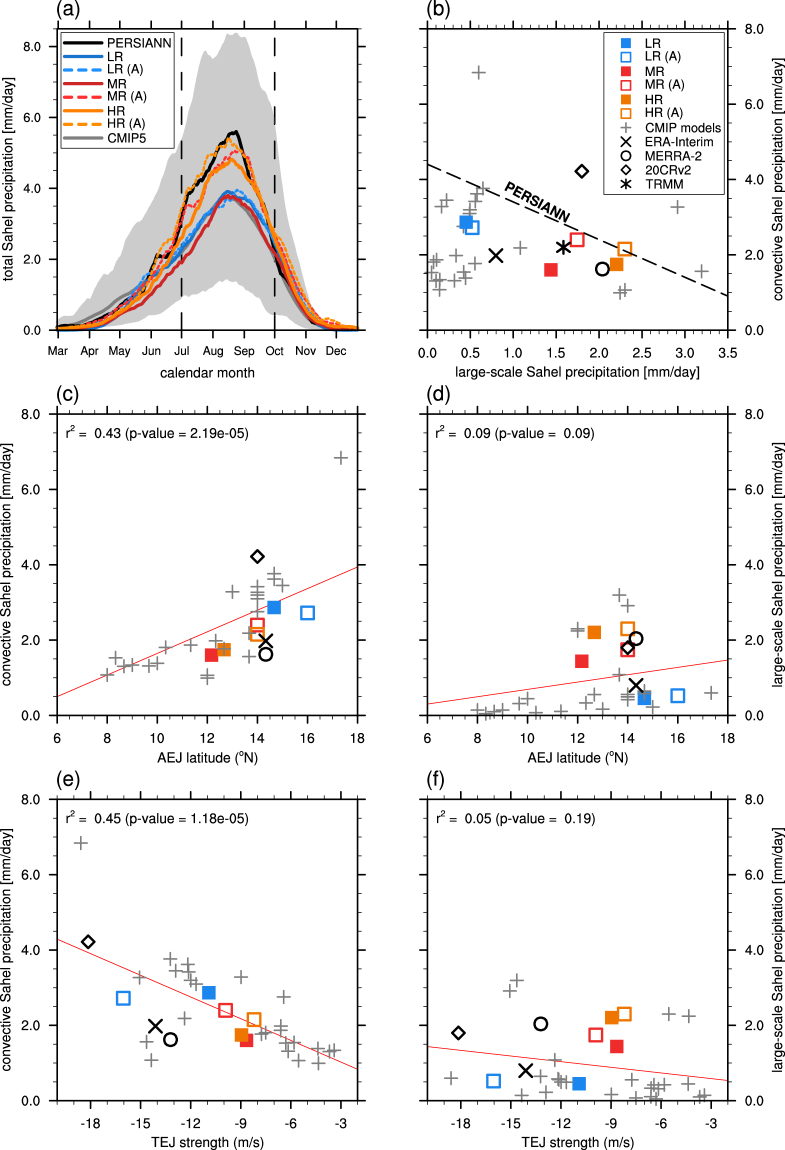
Simulated and observed Sahel rainfall. Coloured lines and symbols represent the coupled KCM simulations with low (LR), medium (MR) and high (HR) model resolution. The corresponding uncoupled integrations are named LR (A), MR (A) and HR (A). Red lines show a simple linear regression model including all 34 data sets together with the corresponding squared Pearson correlation coefficient (r^2^) and the p-value of the null hypothesis using a t-test. All variables are averaged over the domain 20°W-10°E and 10°N–20°N. (**a**) Annual cycle of the daily-mean total rainfall in mm/day. Shading spans the range of all CMIP5 models. Labels indicate the first day of each month. Vertical dashed lines mark July to September (JAS) rainy season. (**b**) Respective contributions of the large-scale and convective precipitation to the JAS total rainfall. Black line shows the observed PERSIANN total precipitation. (**c** and **d**) JAS-mean latitude of the core of the African Easterly Jet (AEJ) versus (**c**) convective and (**d**) large-scale Sahel rainfall in mm/day. (**e** and **f**) JAS-mean strength of the core of the Tropical Easterly Jet (TEJ) versus (**e**) convective and (**f**) large-scale Sahel rainfall in mm/day. The AEJ (TEJ) position is defined as the maximum easterly velocity at the 600 (200) hPa level.

There is a large spread in the annual cycle of total (large-scale plus convective) Sahel rainfall simulated by the CMIP5 models (Fig. 2a). Virtually all models correctly simulate peak rainfall in August, but the magnitudes range from 1 mm/day to 8 mm/day, while observations indicate around 4 mm/day to 6 mm/day. There is a general tendency of the models, including the KCM, to underestimate the rainfall in summer, which is due to a southward displacement of the precipitation maximum (Fig. S1). Although all KCM experiments reproduce the March to May (MAM) precipitation onset reasonably well, they differ in the simulated amplitude during the main monsoon months. A comparison of the coupled simulations with their atmosphere-only counterparts, i.e. forced by the observed SST record and indicated by a trailing (A), allows a separation into resolution and SST bias related differences. Surprisingly, the annual cycle of rainfall in LR and LR (A) is virtually identical, even though LR shows the largest SST biases. This suggests an inherent limitation of the atmospheric component in regard to the moisture advection into West Africa.

Total rainfall in MR is comparable to LR due to a reduction of convective (subgrid-scale) and an increase in large-scale precipitation (Fig. 2b). The amount of large-scale precipitation in the KCM is more sensitive to the atmospheric resolution than to the prevailing SST biases. The spatial pattern of precipitation differences between HR (A) and LR (A) suggests a change of the tropical large-scale precipitation mainly in regions of elevated orography over the African continent (Fig. S2). The finer resolved topographic features in West Africa act to increase the overall large-scale precipitation amount over the coastal regions and the Sahel box. Interestingly TRMM and all reanalysis products also indicate a larger fraction of stratiform rainfall rates of up to 40%, while the CMIP5 models simulate mainly convective precipitation over the Sahel region. The coupled KCM integrations all place the core of the African Easterly Jet (AEJ) further to the south than their uncoupled counterparts, which is associated with a decrease of the convective precipitation for experiments MR and HR (Fig. 2c). The only minor improvements in AEJ location from MR to HR hint at more factors in addition to southeastern tropical SSTs that limit the northward migration of the AEJ. The CMIP5 models support the tendency of reduced convective precipitation over the Sahel for a more southern location of the AEJ. We find a significant correlation that explains 43% of the variability by a simple linear regression (Fig. 2c). The corresponding large-scale precipitation rates also show the lowest overall rates and intermodel spread for AEJ core locations southward of 12°N, but no significant correlation for more northern latitudes (Fig. 2d).

The second higher-level circulation feature that is influencing the WAM is the Tropical Easterly Jet (TEJ) located at a height around 150–200 hPa. In contrast to the AEJ, the TEJ does not influence the total Sahel rainfall amount by its latitudinal position (Fig. S3), but rather by its strength (Fig. 2e). The convective precipitation is again more sensitive to differences in the TEJ than the large-scale fraction (Fig. 2f). We find a significant correlation between a stronger TEJ and associated larger Sahel convective rainfall.

Total Sahel rainfall and its fractionation are close to observations both in MR (A) and HR (A) throughout the year (Fig. 2a and b). While HR shows a similar good performance, MR clearly underestimates total Sahel rainfall. This indicates that the Sahel rainfall in MR is not limited by the atmospheric component, but rather by the flawed simulation of tropical Atlantic SSTs.

**Figure 3:**
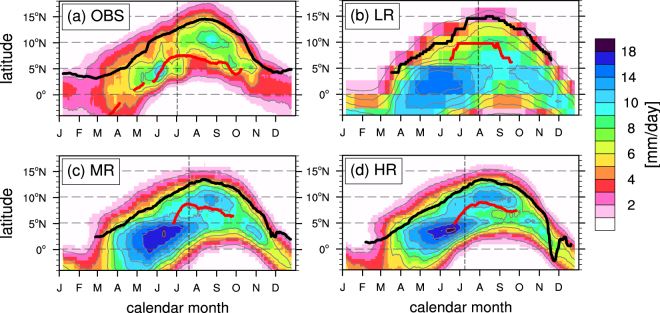
Time-latitude (Hovmöller) diagrams of 14-day smoothed daily precipitation zonally averaged from 10°W to 10°E in mm/day. The thin grey contours have an interval of 2 mm/day. The thick black (red) line indicates the core location of the African (Tropical) Easterly Jet defined as the latitude of maximum eastward velocity at 600 hPa (200 hPa) levels. Locations are only shown for days with core speeds larger than 6 m/s. Observations in (**a**) combine PERSIANN precipitation and ERA-Interim wind data. Results are shown on the individual model grids. The vertical dashed lines show the calculated mean onset dates of the WAM.

The observed seasonal meridional migration of precipitation over West Africa is shown in Fig. 3a. Significant precipitation starts at the coast of the Gulf of Guinea at 5°N around March and increases until it peaks in June. This coastal phase is overestimated in all KCM simulations with a narrowing and intensifying wet bias with increasing resolution. The WAM onset is marked by a distinct “jump” of the precipitation maximum from the coastal region to about 10°N with a mean observed onset day of July 2 and a standard deviation of 9.8 days. The major improvements in MR (Fig. 3c) and HR (Fig. 3d) lead to more realistic simulation of this inland rainfall maximum during JAS, while LR (Fig. 3b) always simulates highest rainfall near the coastline.

The flawed location of the strongest precipitation in LR is caused by a combination of resolution and SST dependent effects. The low atmospheric model resolution itself leads to a dry bias over the Sahel (Fig. S4), while the too warm SSTs in the eastern tropical Atlantic result in excessive precipitation over the Gulf of Guinea and adjacent coastal regions (Fig. S1). The enhanced and earlier development of the ACT during boreal spring and summer in HR leads to an earlier demise of coastal precipitation compared to MR. The mean onset day of July 8 is about 2 weeks (3 weeks) earlier than in MR (LR) and only one week after the observed date. The observed WAM onset is characterized by a sudden reduction of Gulf of Guinea precipitation and a more gradual increase over the Sahel. The precipitation change associated with the onset in HR is distinctly stronger due a stronger northward moisture advection from the coast onto the continent (Fig. S5). All experiments forced by observed SST simulate a too early onset in mid-June, that is mainly caused by a premature maximum and decline of precipitation at 5°N (Fig. S4).

The latitudinal area of strong vertical motion over the Sahel, which is closely linked to total rainfall amounts, is bounded by the TEJ to the south and by the AEJ to the north^7^. The TEJ in all KCM simulations is located further north than in ERA-Interim, which reduces the latitudinal range between TEJ and AEJ and favors the development of a narrower, stronger than observed Sahel rain band in MR and HR. The poleward shift of the TEJ and AEJ in LR leads to a slight increase in rainfall in the northern part of the Sahel during August.

The sudden onset of the WAM in MR and HR indicates an enhanced influence of another climate component on the principally radiatively driven WAM evolution. Caniaux *et al*.^21^ show with an area-based ACT index (hereafter ACT_area_) a significant correlation between the interannual variability of the onset dates of the ACT_area_ and the WAM for the period 1982–2007. For this they defined the ACT_area_ onset as the first day of the year where the area of the equatorial Atlantic with SSTs below 25 °C exceeds 0.4 × 10^6^ km^2^. We also find a significant correlation of 0.66 between anomalies in the WAM onset and the ACT_area_ onset about three weeks earlier (June 10, Fig. S6), despite the different precipitation data and extended time period (1983–2014). This further supports the idea that the ACT dynamics exert a significant influence on the intraseasonal variability of the WAM onset.

**Figure 4:**
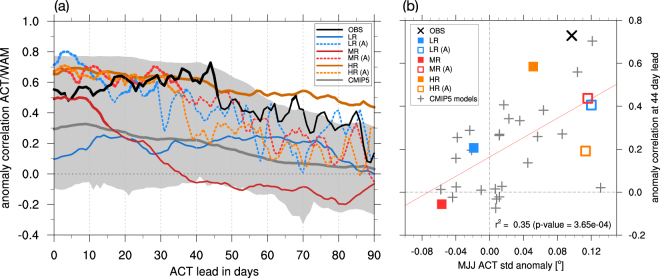
Anomaly correlation as function of the time lag (days) between an Atlantic Cold Tongue (ACT) index (SST anomalies averaged over 20°W-12°E, 5°S-5°N) and the time series of WAM onset date anomalies. Time periods used for the correlation analysis are the years 1983–2014 for the observations, 80 (28) model years for the coupled (uncoupled) KCM integrations and all available years for the CMIP5 models. (**a**) The x-axis shows the number of days by which the ACT index is leading the WAM onset. Thick parts of the lines indicate significance of the Spearman rank order correlation coefficient at the 99% level. Shading indicates the range of all CMIP5 models. (**b**) Scatter plot of the interannual variability of the ACT index (average of months MJJ minus annual-mean) against the anomaly correlation coefficient at a 44 day lead of the ACT index (highest observed correlation). The red line shows a simple linear regression model including all 32 data sets together with the corresponding squared Pearson correlation coefficient (r^2^) and the p-value of the null hypothesis using a t-test.

We now address the question whether this influence is captured in current climate models. Due to the severe SST biases in the ACT region present in most models, simulated ACT_area_ onsets often occur after the WAM onset or do not exceed the threshold value at all (Fig. S6). Therefore, we change from an absolute ACT_area_ onset definition to a simple time series of SST anomalies averaged over the ACT region (hereafter ACT_SST_). A lead-lag correlation analysis with the WAM onset anomalies also yields a correlation of above 0.6 in the observations with the ACT_SST_ leading by three weeks (Fig. 4a). We observe the strongest correlation of 0.73 when ACT_SST_ is leading the WAM onset by 44 days (mid-May). Experiments LR and MR show no, or only modest correlations, while HR is consistent with the observations and simulates ACT_SST_ anomalies that are significantly correlated with the WAM onset anomalies for about 90 days in advance. All uncoupled atmosphere model experiments show high correlations for lead times of up to one month, indicating that the specified SST variability is more important than the atmospheric resolution.

The CMIP5 models show a large spread in anomaly correlation with very low values in the ensemble-mean, but some models feature values comparable to the observations over the whole 90-day period considered. High correlations of WAM onset anomalies with the SST anomalies in the ACT_SST_ region at a 44 day-lead is related to the quality with which interannual SST variability in the ACT_SST_ region is simulated in the models (Fig. 4b). Higher correlation arises from more realistic seasonal phase locking with strong interannual variability in May-July (MJJ), rather than from its magnitude in that season (Fig. S7), expressed as an increases of the explained variance (r^2^) from 23 to 35%. This is the main difference between MR and HR. While the amplitude of the interannual SST variability in the ACT_SST_ region is comparable, it establishes one month earlier in HR than in MR (Fig. S8).

## Discussion and Conclusions

With a set of fully coupled climate model simulations, we show that a more realistic development of the Atlantic Cold Tongue (ACT) significantly improves the seasonal evolution of the West African Monsoon (WAM), and strongly influences the onset of Sahel rainfall and its intraseasonal variability.

The influence of the slowly varying upper tropical Atlantic ocean dynamics on the atmosphere is shown in observational records^21,22^ and potentially allows useful short-term forecasts of the timing of the WAM onset^23^. Vellinga *et al*.^6^ find modest forecast skill in a regional climate model at 2–3 months lead time that derives from tropical Atlantic sea surface temperatures (SSTs) in June. In contrast to our study, these seasonal forecasting systems are initialized with ocean temperatures derived from observations or reanalysis data sets. This means that during their forecast period of several weeks or months these models will normally not experience as severe SST biases as discussed in this paper. In our work we do not assess the seasonal prediction skill in the KCM, but rather show that also multi-year control integrations of global climate models can show robust correlations between the timing of the WAM and equatorial SSTs several weeks or even a few months in advance. Models with less SST bias that simulate an earlier start of the ACT_SST_ formation show a tendency for a longer lasting influence on the WAM. Moreover, in the case of an overall too late or too weak ACT_SST_ the precipitation over the Sahel shows a more gradual progression following the solar insolation maximum. The improvements shown for the KCM can eventually lead to a better representation of the mean state in future versions of regional forecast models or could be used to identify changes between the SST-WAM coupling under future warming scenarios in global climate model simulations.

All KCM and CMIP5 simulations show a delayed onset and smaller extent of the Atlantic cold tongue during boreal spring and summer compared to the observations. The associated warmer sea surface temperatures in the simulations are generally linked to higher rates of marine precipitation in the Gulf of Guinea. A comparison of the coupled and uncoupled simulations shows that this is the main reason for the coastal wet bias in the KCM configurations during July to September. The earlier and stronger cold tongue development in HR and the resulting lower surface temperatures greatly reduce the bias in the Gulf of Guinea compared to LR and MR. The resulting earlier demise of coastal precipitation and shift of the precipitation maximum into the Sahel in HR leads to a WAM onset that is three weeks earlier than in LR and very close to the observed one. The three simulations show the dominant influence of southeastern and equatorial SSTs on the strength of coastal precipitation and timing of the WAM onset in the KCM.

Sufficiently high atmosphere model resolution is a key in the KCM to reduce biases in the tropical Atlantic sector mean climate and seasonal to interannual variability, in particular with regard to Sahel rainfall. This has been shown by a set of integrations of the KCM with varying atmospheric but fixed oceanic resolution. With regard to that model, Harlaß *et al*.^18,19^ find that the vertical atmosphere model resolution is of particular importance for tropical Atlantic interannual variability. Whether these promising results can help to improve tropical climate simulations in other, especially non-spectral atmospheric models needs to be tested in the future.

We still see significant biases even in the highest-resolution version of the KCM (HR, T255L62), which not only are reflected in the tropical Atlantic SST but also in a too narrow, slightly southward displaced precipitation maximum over West Africa. Nevertheless, there is a strong link between the interannual variability of the ACT and WAM onset in the HR-version. We are therefore optimistic that models depicting reasonable ACT/WAM correlation, including some of the CMIP5 models, can be successfully used to forecast WAM onset at lead times of several weeks even in the presence of mean state biases. Furthermore, Richter *et al*.^12^ describe some CMIP5 models which, in contrast to most CMIP3 models, do reasonably well in reproducing key features of the Atlantic zonal mode.

In the set of KCM simulations, the influence of the reduced SST bias in boreal summer is mainly expressed as a shift of the rainfall maximum from the Gulf of Guinea in the eastern part of the tropical Atlantic in the low-atmospheric resolution version (LR) towards the central and western parts of the basin at medium (MR) and high (HR) atmospheric resolution. Eichhorn and Bader^13^ find a similar influence of the SST bias in an idealized study with ECHAM6, the successor to the atmosphere model used in the KCM. They further show how this westward shift is associated with an increase of easterly trade winds and moisture advection along the equator.

The tropical Atlantic SST biases affect Sahel rainfall mainly by a southward displacement of the monsoonal system, expressed by the African Easterly Jet (AEJ) and Tropical Easterly Jet (TEJ) core locations. The AEJ latitude for all atmosphere model versions used here is similar to the reanalysis values, when forced by observed SSTs. The coupled versions all place the AEJ farther south, leading to reduced rates of convective precipitation over the Sahel. The influence of the SST biases in the southeastern and northeastern tropical Atlantic is increased from LR to MR due to the enhanced cold SST bias off Senegal and Mauritania that limits the northward migration of the Intertropical Convergence Zone (ITCZ). This problem, however, is no longer present in HR. The strong relation between the TEJ and convective precipitation over the Sahel found here has been documented in previous studies about the interannual variability of the WAM^7,24^. The main mechanism linking both is described as an increase in upper-level divergence resulting from the strong meridional components associated with the TEJ^25^ that ultimately favour deep convective motion over West Africa.

The Sahel rainfall in the KCM not only is affected by the flawed representation of tropical Atlantic SSTs but also by inherent limitations of the atmospheric model component itself. Even when forced by observed SSTs the low-resolution configuration LR (A) is not able to transport enough moisture onto the West African continent and produce sufficient Sahel rainfall. All atmosphere-only runs place the precipitation maximum further to the western tropical Atlantic than observed. The magnitude of the wet bias even increases from LR (A) to MR (A) and to HR (A). Siongco *et al*.^26^ show that current atmosphere models can be divided into two groups based on their Atlantic maximum precipitation being located either near the coast of Brazil or the coast of Guinea, and that this grouping already originates in boreal spring. We hypothesize that the moisture advection into the Sahel in LR is strongly limited by the low-resolution atmosphere model and therefore less sensitive to tropical SSTs than MR and HR.

Another obvious shortcoming is the excessive precipitation during May and June around the Gulf of Guinea visible in all KCM simulations. This overestimation of the ITCZ strength gets even amplified with increasing atmospheric resolution. We find the same model behavior for the atmosphere-only configurations and therefore attribute it to an internal model limitation rather than to flawed boundary conditions. Other studies also find a general intensification of tropical ITCZ^27^ or a strengthening of the East Atlantic bias^26^ with increasing model resolution. Siongco *et al*.^26^ demonstrate for two different models how the increased resolution enhances low-level (850 hPa) westerlies that suppress precipitation over the Brazil coast and lead to increased convergence and convective rainfall over the Gulf of Guinea. Harlaß *et al*.^19^ find that the vertical motions and rainfall north of the equator strengthen with increased resolution by enhanced southerly advection of zonal momentum. We further speculate that the existing cloud and convection parameterizations were developed for lower model resolutions and maybe need additional testing and tuning to reduce the overly strong ITCZ at higher atmospheric resolutions.

We want to emphasize that area-averaged indices, like the Sahel index used here, always allow that model deficits are masked by error compensation. The very good agreement in the total precipitation Sahel index between HR and the observations is partly caused by counteracting effects of a wet bias of the large-scale precipitation in the southern part of the Sahel box and a dry bias of the convective precipitation in the north. The resulting coastal precipitation is higher than in HR (A) throughout the WAM season and therefore leads to later mean WAM onset date. In fact, the mean onset day in HR (July 8) is closer to observations (July 2) than in the corresponding atmosphere-only simulation (June 13).

## Methods

### Kiel Climate Model

We employ the Kiel Climate Model^28^ a fully coupled atmosphere-ocean-sea ice general circulation model. The ocean component NEMO^29^ is kept fixed for all integrations at a horizontal resolution of 2° with a latitudinal refinement to 0.5° close to the equator and 31 vertical levels. We use three different configurations of the ECHAM5 spectral atmospheric general circulation model^30^. The low-resolution version (hereafter “LR”) is run at a horizontal resolution of ~2.8° (T42, 31 vertical levels), while both the horizontal and vertical resolution are increased for the medium-resolution version (hereafter “MR”) at ~0.75° (T159) and 62 levels. A further increase of the horizontal resolution (T255, ~0.47°, 62 levels) is used for the high-resolution version (hereafter “HR”). The top level is the same at about 5 hPa in all three configurations. Additional levels are placed in between the 31 levels and concentrate towards the surface. Parametrization schemes, as for example cloud microphysics and optical properties or cumulus convection, are scale-aware in ECHAM5^30^ and vary with resolution but are not re-tuned in any way. Results are confined to the last 80 years of 100 years long integrations.

We additionally integrate all three atmosphere model versions in uncoupled mode under the same conditions, forced by observed SST for the period 1982–2009 to identify differences solely caused by the changes in atmospheric resolution. We will refer to these atmosphere-only experiments as LR (A), MR (A) and HR (A) respectively.

### CMIP5

For comparison, we also analyze model data from phase 5 of the Coupled Model Intercomparison Project (CMIP5). We include in our analyses all models providing daily mean data for the “historical” experiment with the observed atmospheric composition changes^31^, available at the time of the analysis. No model selection in any form was done. An overview of the 25 analysed models is given in Table TS1.

### Data

Observational data sets used for comparison include the NOAA 1/4° daily “Optimum Interpolation Sea Surface Temperature” (OISST) version 2^32^ and the “Precipitation Estimation from Remotely Sensed Information using Artificial Neural Networks-Climate Data Record” PERSIANN-CDR^33^. Both provide high-resolution (0.25°), daily-mean data for a long overlapping time series from 1983-present and therefore allow a more robust assessment of their interannual variability. We also use the data product 3A25 from the Tropical Rainfall Measuring Mission TRMM^34^ that provide a fractionation of total rainfall rates into their convective and stratiform contributions for the time period 1998–2013. The rain type classification in TRMM is designated as the algorithm 2A-23. The algorithm uses precipitation radar measurements and two different methods (V-method and H-method) to classify precipitation at each grid point into the three categories stratiform, convective and other. Details on the method can be found in Awaka *et al*.^35^. Reanalysis data for the time period 1983–2015 are additionally used and obtained from ERA-Interim^36^, MERRA-2^37^ and the Twentieth Century Reanalysis Project V2^38^. All analyzed observational and model data are interpolated to a global 1° grid prior to analysis.

### Monsoon onset

Many definitions have been used in the literature to quantify the WAM onset date^39^, i.e. the rapid latitudinal shift of the precipitation maximum from 5°N to about 10°N at the end of June/early July. To capture the large-scale shift of the precipitation band we closely follow the approach from Fontaine and Louvet^40^ and Vellinga *et al*.^6^ and define two rainfall indices of zonally averaged precipitation between 10°W to 10°E, one southern index from the equator to 7.5°N and a northern index from 7.5°N to 20°N. Both indices are smoothed with a 15-day low-pass filter and normalized by their respective standard deviations to reduce the influence of mean-state biases in the coupled models. The onset is defined as the first day after 1 June where the northern index exceeds the southern index for at least 15 consecutive days, or for the longest period in years that have no clear onset.

## Electronic supplementary material

Supplementary Information
