# Author Correction: Sahel rainfall strength and onset improvements due to more realistic Atlantic cold tongue development in a climate model

**DOI:** 10.1038/s41598-019-42950-z

**Published:** 2019-05-02

**Authors:** S. Steinig, J. Harlaß, W. Park, M. Latif

**Affiliations:** 1 GEOMAR Helmholtz Centre for Ocean Research Kiel, Wischhofstr. 1-3, 24148 Kiel, Germany; 2 Kiel University, Kiel, Germany

Correction to: *Scientific Reports* 10.1038/s41598-018-20904-1, published online 07 February 2018

This Article contains errors.

In the Introduction,

“The maximum continental warming follows the solar heating northward, whereas the maximum cooling of the ocean surface, driven by meridional and zonal wind stress, is geographically fixed just south of the Equator. These different physical processes combined with land-air interactions^3,4^ lead to a more step-wise than gradual onset of the WAM^5^.”

should read:

“The maximum continental warming follows the solar heating northward, whereas the wind-driven maximum cooling of the ocean surface remains south of the Equator^5^. These different physical processes combined with land-air interactions^3,4^ lead to a more step-wise than gradual onset of the WAM.”

In the legend of Figure 1,

“SST biases against OISSTv2 for the months July to August.”

should read:

“SST biases against OISSTv2 for the months July to September.”

In the Acknowledgements,

“We acknowledge the World Climate Research Programme’s Working Group on Coupled Modelling, the climate modeling groups (listed in Table TS1 in the supplementary information), ECMWF, NOAA and NASA for providing the model output and data sets. This work was supported by the Bundesministerium für Bildung und Forschung grant SACUS (03G0837A) and EU FP7/2007–2013 under Grant agreement no. 603521, project PREFACE. Model integrations were performed at the Norddeutscher Verbund für Hoch- und Höchstleistungsrechnen and the Rechenzentrum der Universität Kiel.”

should read:

“We thank Christian Wengel for carrying out the high-resolution uncoupled atmospheric integrations. We acknowledge the World Climate Research Programme’s Working Group on Coupled Modelling, the climate modeling groups (listed in Table TS1 in the supplementary information), ECMWF, NOAA and NASA for providing the model output and data sets. This work was supported by the Bundesministerium für Bildung und Forschung grant SACUS (03G0837A) and EU FP7/2007–2013 under Grant agreement no. 603521, project PREFACE. Model integrations were performed at the Norddeutscher Verbund für Hoch- und Höchstleistungsrechnen and the Rechenzentrum der Universität Kiel.”

