# Supplementary material for: Sahel rainfall strength and onset improvements due to more realistic Atlantic cold tongue development in a climate model
Source: Sci Rep. 2018 Feb 7;8:2569. doi: 10.1038/s41598-018-20904-1 (PMC5803197; doi:10.1038/s41598-018-20904-1)
Supplement: Supplementary file 1 — Supplementary Information [file 41598_2018_20904_MOESM1_ESM.pdf]

Supplementary information for manuscript:

Sahel rainfall strength and onset improvements due to more realistic Atlantic cold tongue development in a climate model

[1*]Steinig, S., [1]Harlaß, J., [1]Park, W., and [1,2]Latif, M

[1]GEOMAR

Helmholtz Centre for Ocean Research Kiel

Wischhofstr. 1-3

24148 Kiel

Germany

[2]Ecxellence Cluster “The Future Ocean”

University of Kiel

Christian-Albrechts-Platz 4, 24118 Kiel

Germany

*Corresponding author: S. Steinig (ssteinig@geomar.de)

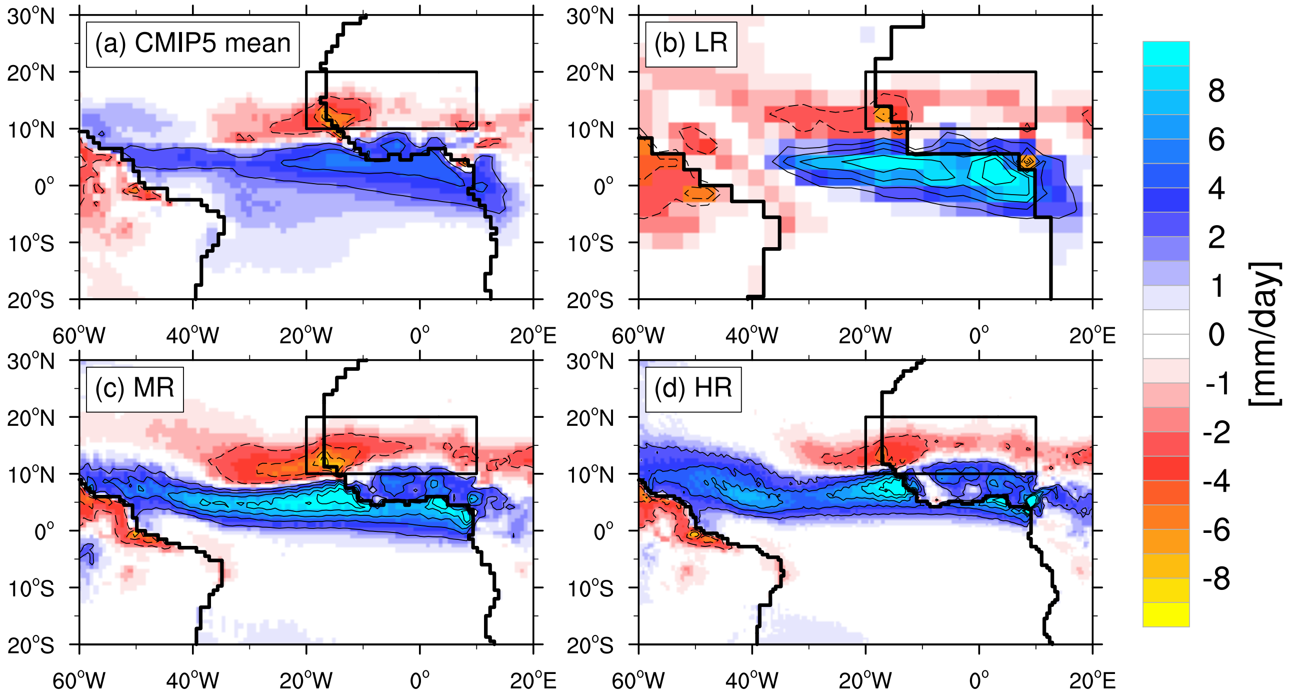

Figure S1. Total precipitation model biases against PERSIANN-CDR for the months JAS. Values are in mm/day with an interval of 2 mm/day for the black contour lines. (a) CMIP5 ensemble mean for the years 1982-2005, (b) LR (T42L31), (c) MR (T159L62) and (d) HR (T255L62). Black rectangle indicates the domain 20°W-10°E and 10°N-20°N used for calculation of the Sahel mean values in Figure 2. Maps are created with the NCAR Command Language (Version 6.4.0) [Software]. (2017). Boulder, Colorado: UCAR/NCAR/CISL/TDD. http://dx.doi.org/10.5065/D6WD3XH5

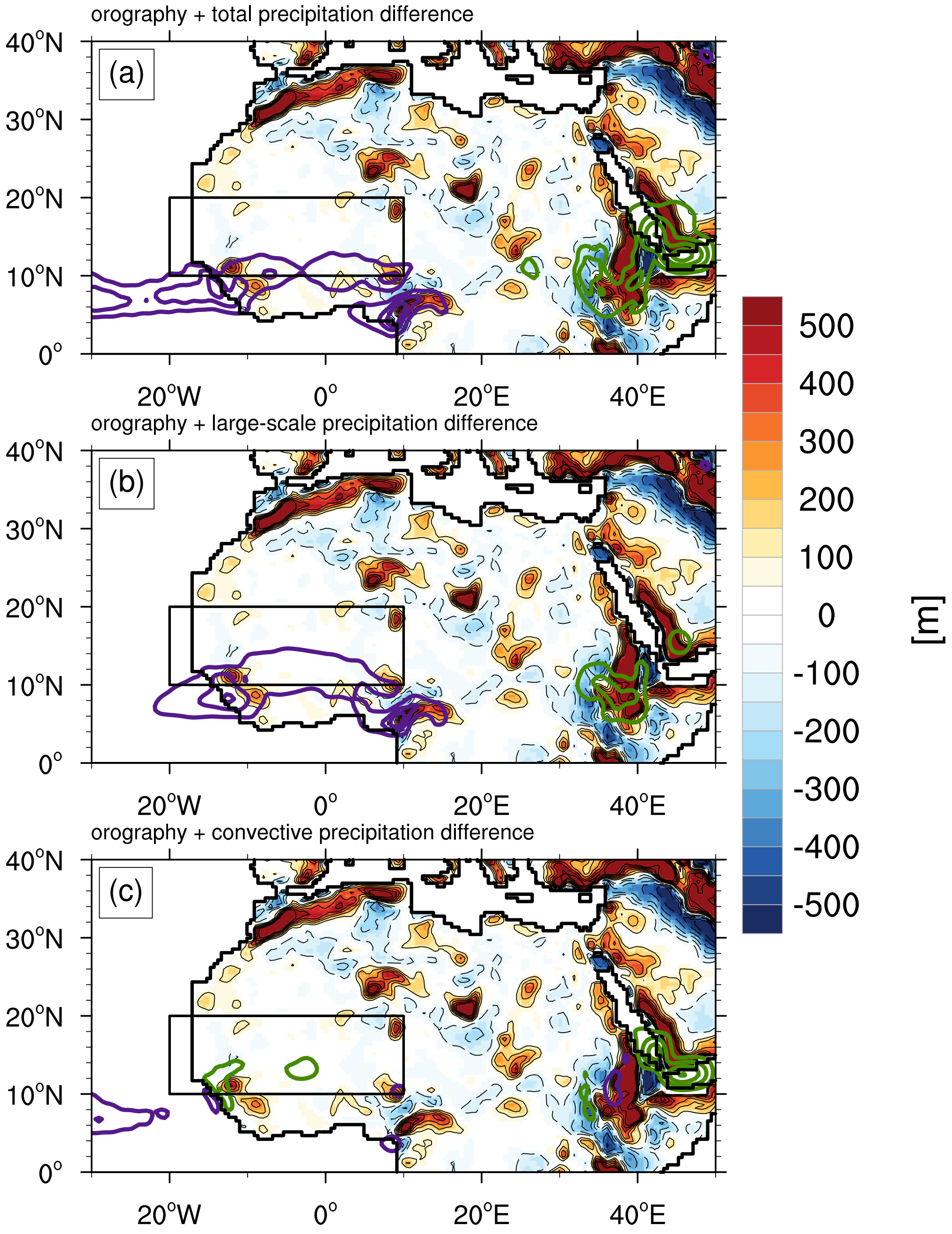

Figure S2. Differences in surface elevation (shading; in m) and July to September precipitation (contour interval 2 mm/day; purple (green) indicating wetter (dryer) conditions) between the atmosphere-only experiments HR (A) minus LR (A). Differences in total precipitation (a) are nearly completely caused by difference in the large-scale precipitation (b) and highest around regions of increased topography. Differences in convective precipitation (c) are small over West Africa. Maps are created with the NCAR Command Language (Version 6.4.0) [Software]. (2017). Boulder, Colorado: UCAR/NCAR/CISL/TDD. http://dx.doi.org/10.5065/D6WD3XH5

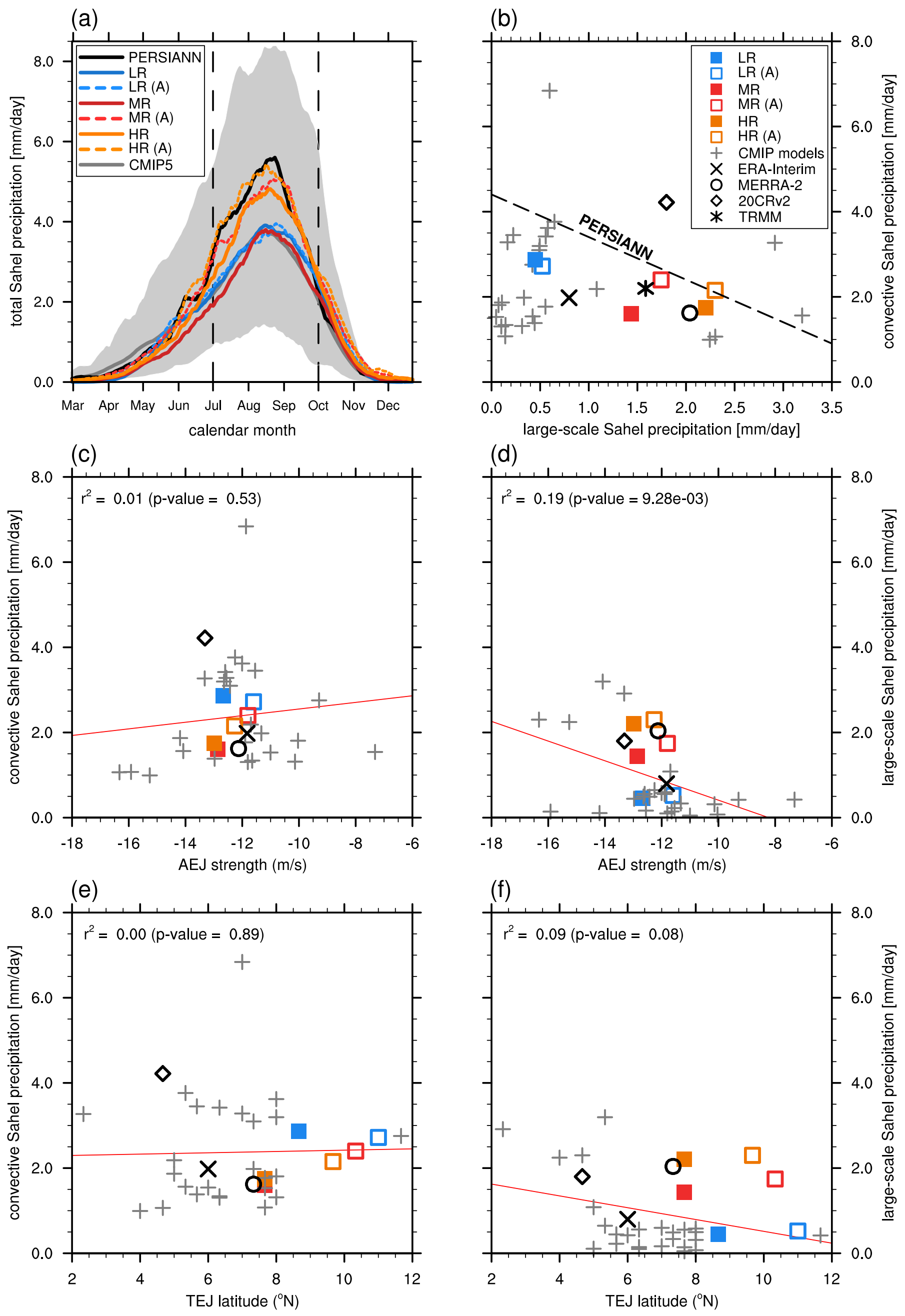

Figure S3. Simulated and observed Sahel rainfall. Coloured lines and symbols represent the coupled KCM simulations with low (LR), medium (MR) and high (HR) model resolution. The corresponding uncoupled integrations are named LR (A), MR (A) and HR (A). Red lines show a simple linear regression model including all 34 data sets together with the corresponding squared Pearson correlation coefficient (r^2) and the p-value of the null hypothesis using a t-test. All variables are averaged over the domain 20°W-10°E and 10°N-20°N. (a) Annual cycle of the daily-mean total rainfall in mm/day. Shading spans the range of all CMIP5 models. Labels indicate the first day of each month. Vertical dashed lines mark July to September (JAS) rainy season. (b) Respective contributions of the large-scale and convective precipitation to the JAS total rainfall. Black line shows the observed PERSIANN total precipitation. (c)-(d) JAS-mean strength of the core of the African Easterly Jet (AEJ) versus (c) convective and (d) large-scale Sahel rainfall in mm/day. (e)-(f) JAS-mean latitude of the core of the Tropical Easterly Jet (TEJ) versus (e) convective and (f) large-scale Sahel rainfall in mm/day. The AEJ (TEJ) position is defined as the maximum easterly velocity at the 600 (200) hPa level.

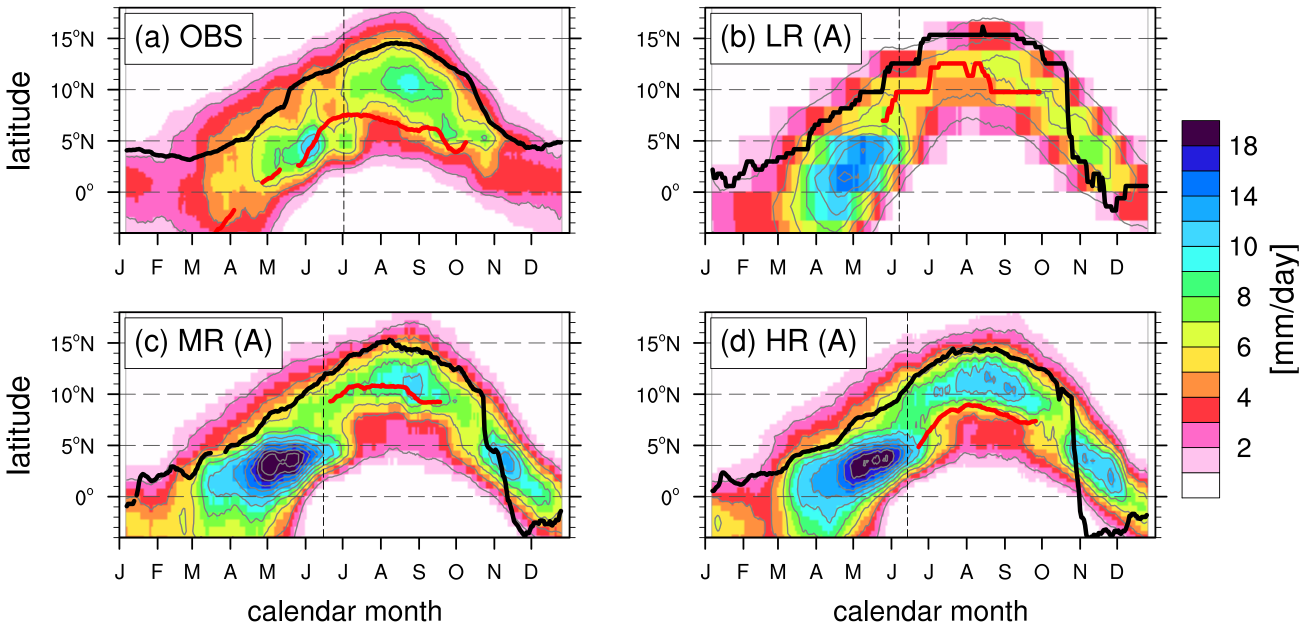

Figure S4. Time-latitude (Hovmöller) diagrams of 14-day smoothed daily precipitation zonally averaged from 10°W to 10°E in mm/day. The thin grey contours have an interval of 2 mm/day. The thick black (red) line indicates the core location of the African (Tropical) Easterly Jet defined as the latitude of maximum eastward velocity at 600 hPa (200 hPa) levels. Locations are only shown for days with core speeds larger than 6 m/s. Observations in (a) combine PERSIANN precipitation and ERA-Interim wind data. Results are shown on the individual model grids. The vertical dashed lines show the calculated mean onset dates of the WAM. This figure is comparable to Fig. 3 of the main manuscript, but shows the corresponding AGCM counterparts.

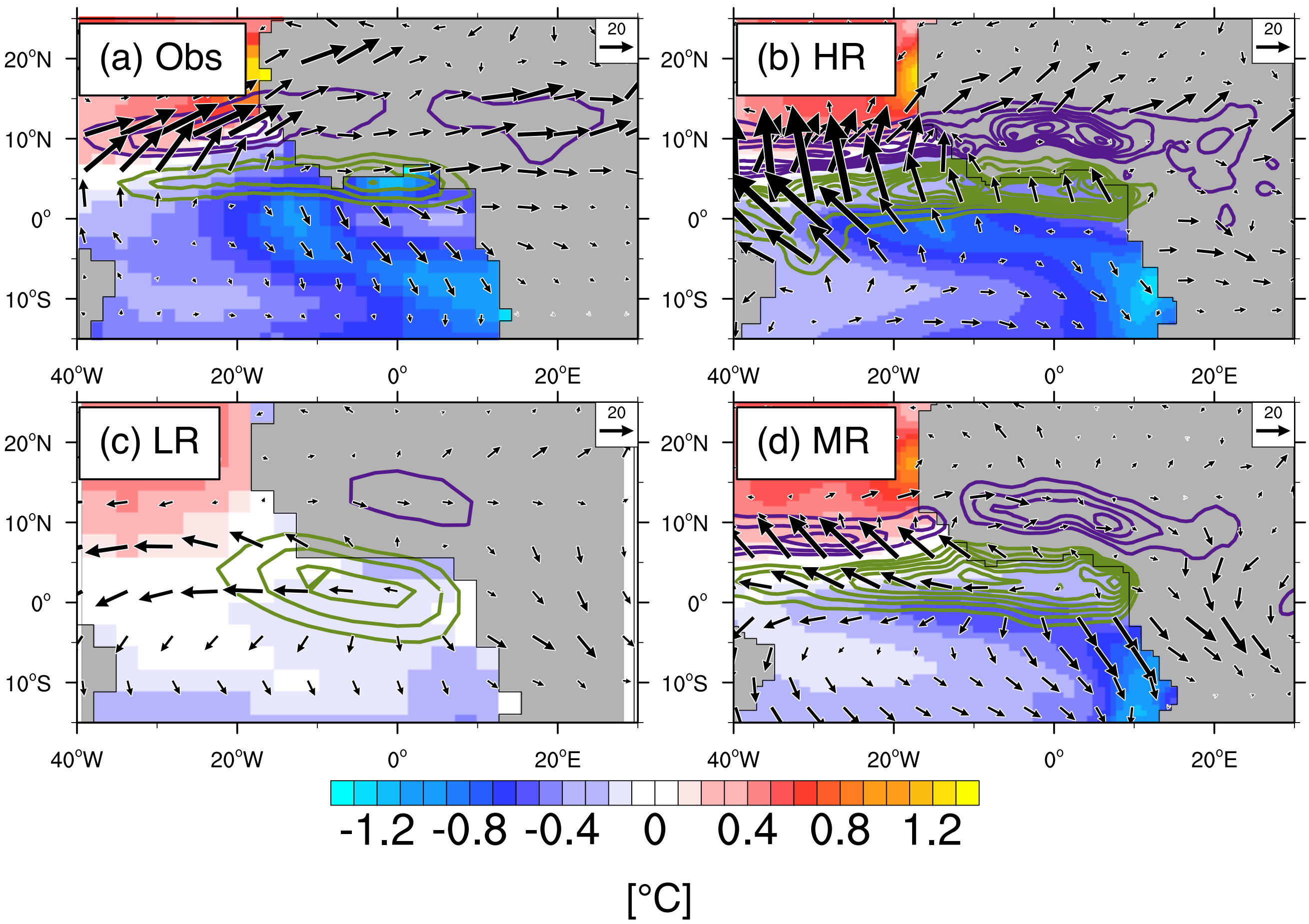

Figure S5. Modelled and observed climatic change associated with the onset of the WAM. Composite fields are shifted around their calculated onset date for each year and are shown as the difference of the 15 days after the onset minus the 15 days before the onset. Shading represents SST in °C, contour lines precipitation in mm/day and arrows the atmospheric moisture flux integrated from the bottom to the 850 hPa layer in kg/m/s relative to the reference vector length in the upper right corner. Contour line interval is 1 mm/day with green (purple) lines indicating dryer (wetter) conditions. Observational data includes OISST, PERSIANN and ERA-Interim winds. Maps are created with the NCAR Command Language (Version 6.4.0) [Software]. (2017). Boulder, Colorado: UCAR/NCAR/CISL/TDD. http://dx.doi.org/10.5065/D6WD3XH5

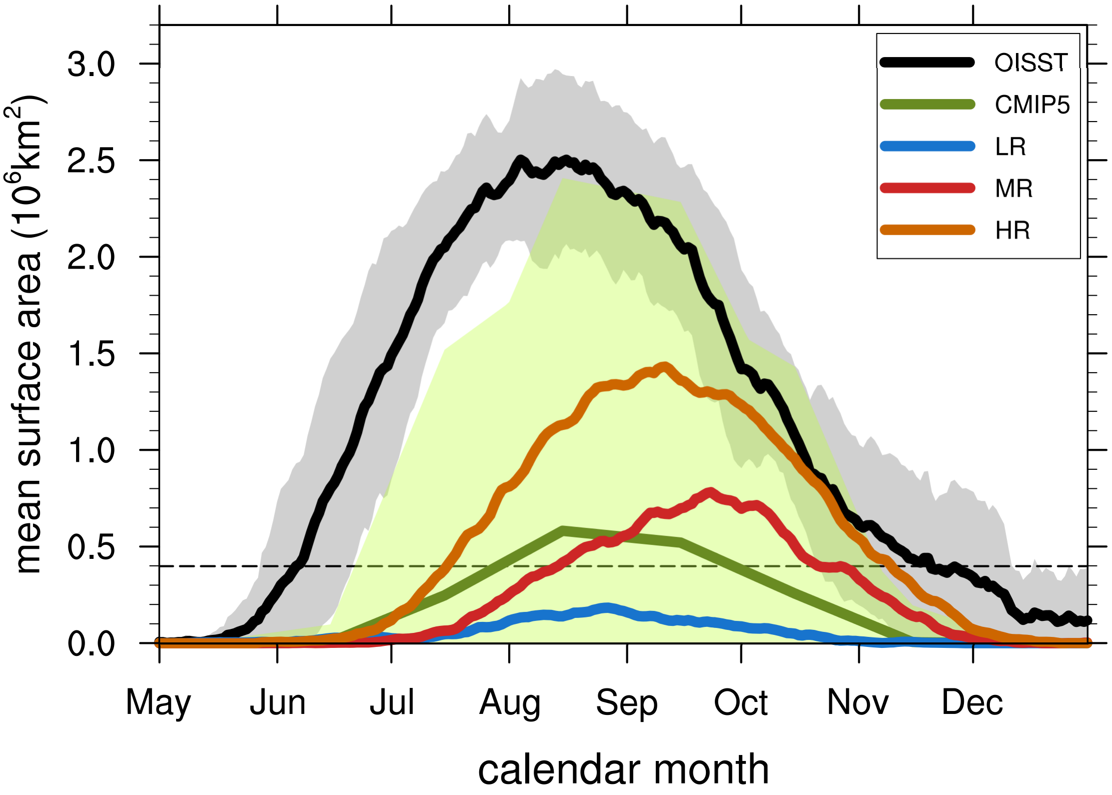

Figure S6. Seasonal evolution of the ACT surface area following the procedure by Caniaux et al. (2011). Shown is the cumulated surface area in the region 20°W-12°E, 5°S-5°N with absolute SSTs below 25°C. We slightly reduce the westward extension of our area to eliminate the influence of erroneously low SSTs in the western equatorial Atlantic arising in most model simulations. Gray shading indicates plus and minus one standard deviation of the observed interannual variability. Green shading represents the range of all CMIP5 models. Time labels show the first day of each month.

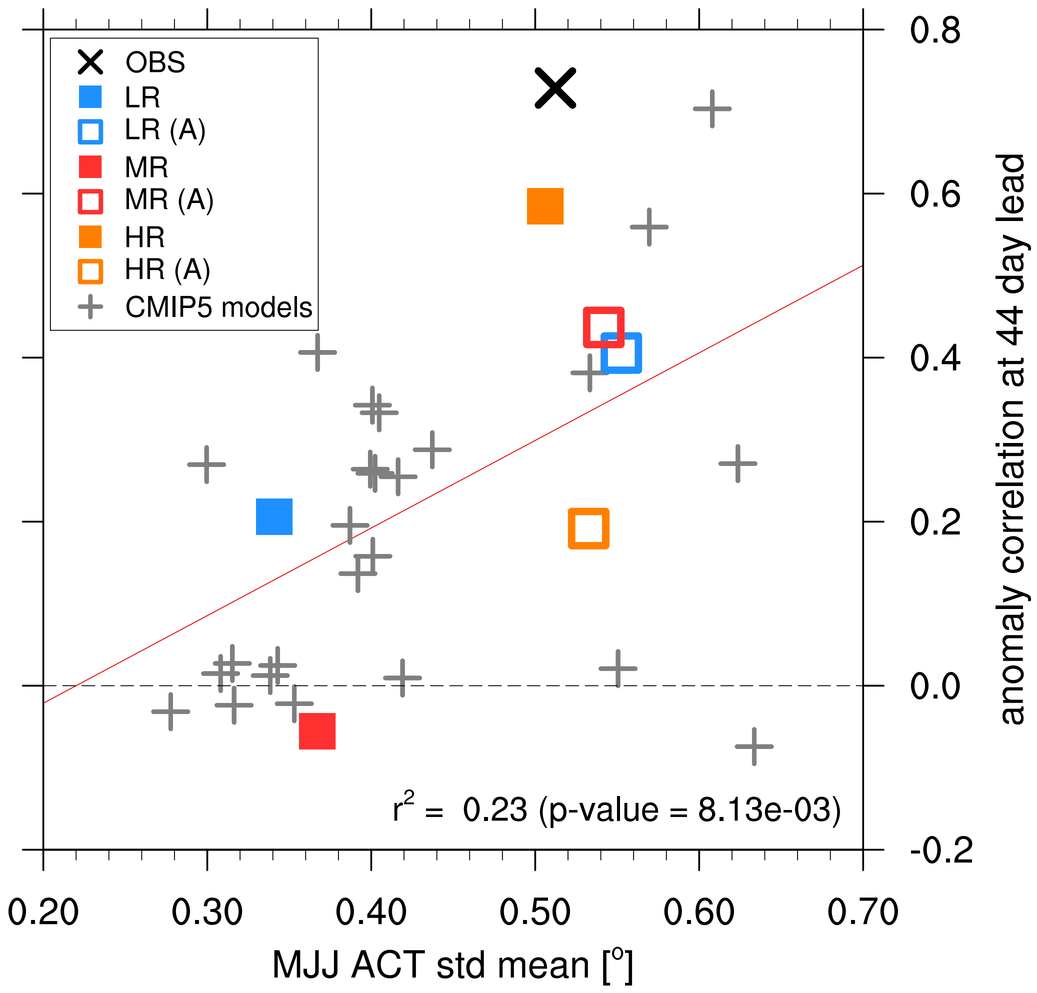

Figure S7. Reproduction of Figure 4b, but showing the absolute strength of the ACT interannual variability averaged over the months MJJ, rather than the difference to the annual mean.

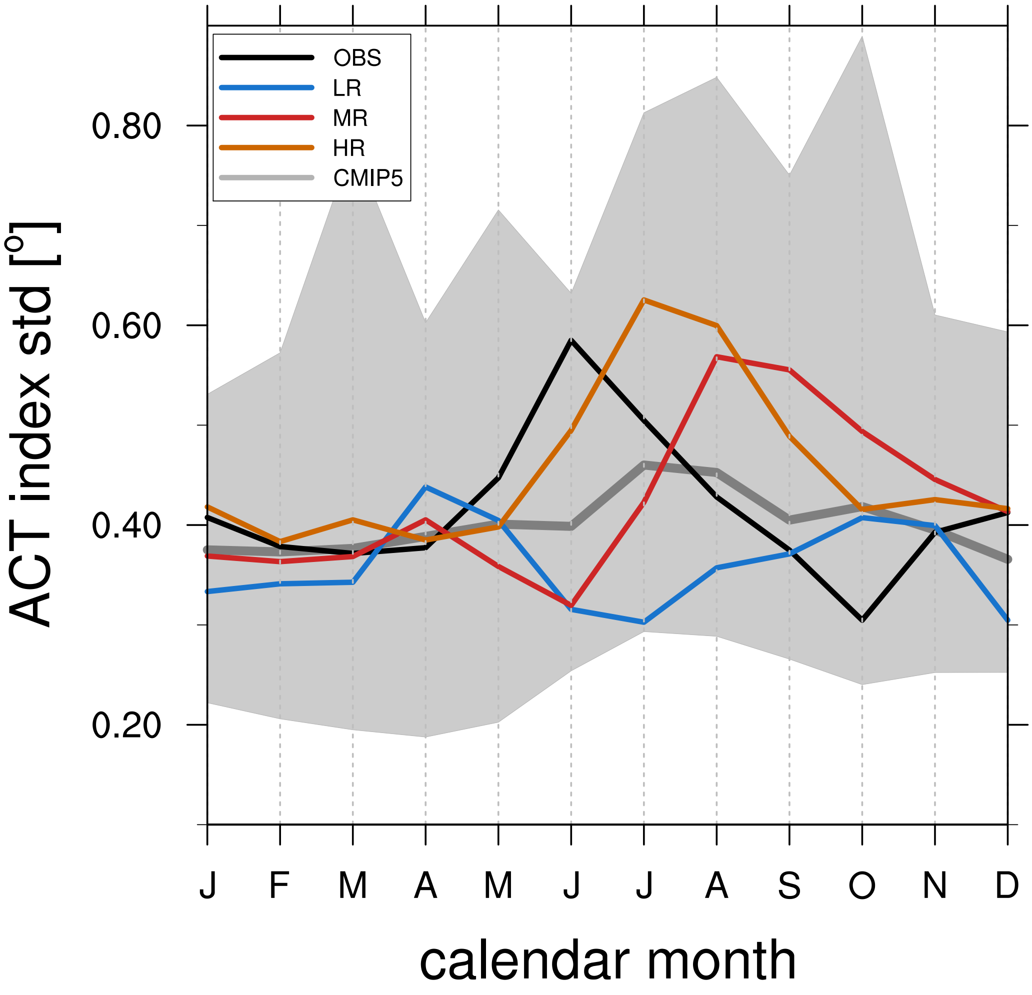

Figure S8. Monthly mean interannual standard deviation for SST anomalies in °C averaged over the ACT region (20°W-12°E, 5°S-5°N). Shading indicates the range of all CMIP5 models.

Modelling Centre (or Group)	Institute ID	Model Name
Commonwealth Scientific and Industrial Research Organization (CSIRO) and Bureau of Meteorology (BOM), Australia	CSIRO-BOM	ACCESS1.0
Commonwealth Scientific and Industrial Research Organization (CSIRO) and Bureau of Meteorology (BOM), Australia	CSIRO-BOM	ACCESS1.3
Canadian Centre for Climate Modelling and Analysis	CCCMA	CanESM2
National Center for Atmospheric Research	NCAR	CCSM4
Centro Euro-Mediterraneo per I Cambiamenti Climatici	CMCC	CMCC-CM
Centro Euro-Mediterraneo per I Cambiamenti Climatici	CMCC	CMCC-CMS
Centre National de Recherches Météorologiques / Centre Européen de Recherche et Formation Avancée en Calcul Scientifique	CNRM-CERFACS	CNRM-CM5
NOAA Geophysical Fluid Dynamics Laboratory	NOAA GFDL	GFDL-CM3
NOAA Geophysical Fluid Dynamics Laboratory	NOAA GFDL	GFDL-ESM2G
Met Office Hadley Centre	MOHC	HadCM3
Met Office Hadley Centre	MOHC	HadGEM2-CC
Met Office Hadley Centre	MOHC	HadGEM2-ES
Institute for Numerical Mathematics	INM	INM-CM4
Institut Pierre-Simon Laplace	IPSL	IPSL-CM5A-LR
Institut Pierre-Simon Laplace	IPSL	IPSL-CM5A-MR
Institut Pierre-Simon Laplace	IPSL	IPSL-CM5B-LR
Atmosphere and Ocean Research Institute (The University of Tokyo), National Institute for Environmental Studies, and Japan Agency for Marine-Earth Science and Technology	MIROC	MIROC4h
Atmosphere and Ocean Research Institute (The University of Tokyo), National Institute for Environmental Studies, and Japan Agency for Marine-Earth Science and Technology	MIROC	MIROC5
Japan Agency for Marine-Earth Science and Technology, Atmosphere and Ocean Research Institute (The University of Tokyo), and National Institute for Environmental Studies	MIROC	MIROC-ESM
Japan Agency for Marine-Earth Science and Technology, Atmosphere and Ocean Research Institute (The University of Tokyo), and National Institute for Environmental Studies	MIROC	MIROC-ESM-CHEM
Max Planck Institute for Meteorology	MPI-M	MPI-ESM-LR
Max Planck Institute for Meteorology	MPI-M	MPI-ESM-MR
Max Planck Institute for Meteorology	MPI-M	MPI-ESM-P
Meteorological Research Institute	MRI	MRI-CGCM3
Meteorological Research Institute	MRI	MRI-ESM1

Table TS1. CMIP5 models and their associated modelling centres or groups used in this study. All models that provided both daily mean data of precipitation, sea surface temperature and monthly mean data of zonal wind at the time of the analysis were used in this study.
